# The Role of “Hierarchical and Classified Prevention and Control Measures (HCPC)” Strategy for SARS-CoV-2 Delta Variant in Guangzhou: A Modeling Study

**DOI:** 10.1007/s44197-023-00108-1

**Published:** 2023-05-31

**Authors:** Yu Ma, Hui Wang, Yong Huang, Chun Chen, Shihao Liang, Mengmeng Ma, Xinjun He, Kangning Cai, Zengtao Jiao, Liyi Chen, Bowei Zhu, Ke Li, Chaojun Xie, Lei Luo, Zhoubin Zhang

**Affiliations:** 1 Guangzhou Center for Disease Control and Prevention, Guangzhou, Guangdong 510120 People’s Republic of China; 2 Institute of Public Health, Guangzhou Medical University & Guangzhou Center for Disease Control and Prevention, Guangzhou, Guangdong 510120 People’s Republic of China; 3 Present Address: Yidu Cloud (Beijing) Technology Co.Ltd, Beijing, 100083 People’s Republic of China; 4 Guangzhou Huadu District Center for Disease Control and Prevention, Guangzhou, China

**Keywords:** COVID-19, Model simulation, SARS-CoV-2, Non-pharmaceutical intervention

## Abstract

**Background:**

The Delta variant of SARS-COV-2 has replaced previously circulating strains around the world in 2021. Sporadic outbreaks of the Delta variant in China have posed a concern about how to properly respond to the battle against evolving COVID-19. Here, we analyzed the “hierarchical and classified prevention and control (HCPC)” measures strategy deployed during the recent Guangzhou outbreak.

**Methods:**

A modified susceptible–exposed–pre-symptomatic–infectious–recovered (SEPIR) model was developed and applied to study a range of different scenarios to evaluate the effectiveness of policy deployment. We simulated severe different scenarios to understand policy implementation and timing of implementation. Two outcomes were measured: magnitude of transmission and duration of transmission. The outcomes of scenario evaluations were presented relative to the reality case (i.e., 368 cases in 34 days) with 95% confidence interval (CI).

**Results:**

Based on our simulation, the outbreak would become out of control with 7 million estimated infections under the assumption of the absence of any interventions than the 153 reported cases in reality in Guangzhou. The simulation on delayed implementation of interventions showed that the total case numbers would also increase by 166.67%–813.07% if the interventions were delayed by 3 days or 7 days.

**Conclusions:**

It may be concluded that timely and more precise interventions including mass testing and graded community management are effective measures for Delta variant containment in China.

## Backgroud

The Delta variant (B.1.617.2) of SARS-CoV-2 was first identified in October 2020 in India with its spike protein containing a set of signature mutations: T19R, (G142D), 156del, 157del, R158G, L452R, T478K, D614G, P681R, and D950N [1–3]. Campbell et al*.* concluded that the Delta variant transmits faster than previously reported variants of concerns (VOCs) including the World Health Organization-designated Alpha, Beta and Gamma variants based on the analysis of the GISAID genomic sequences of the SARS-CoV-2 [4]. Since the discovery of the Delta variant, about 44% of the infections in the UK and 39% in India were attributed to the Delta variant [5]. The emergence of the Delta variant, the population density, the social behavioral phenomena such as participation in large gatherings, and/or the lack of preventive measures resulted in the surge in COVID-19 cases in India [6]. On 17 May 2021, the UK government decided to drop the requirement to wear masks in schools, and the data for the week to 29 May from the Office for National Statistics showed that the numbers of COVID-19 cases had risen fastest in schoolchildren in years [7]. It is suggested that the Delta variant has replaced the other circulating variants including the previously more contagious Alpha variant (B.1.1.7) and become a major concern of the worldwide COVID-19 pandemic currently [4, 8, 9].

Since the identification of the Delta variant, there were reports on sporadically imported cases infected with this variant in China in Chongqing [10], Guangxi [11], and Guangdong [12]. The Guangzhou cluster is particularly the first big cluster with community transmission so far, which started with the identification of a domestic case of COVID-19 on May 21st and continued to June 18th, 2021, with 153 total reported cases and several generations of transmission in Guangzhou. Before the prevalence of Delta mutant strains, China mainly adopted three major groups of NPI measures, including inter-city travel restrictions, early identification and isolation of cases, contact restrictions and social distancing measures, and achieved good prevention and control results in rapidly controlling the scale of the epidemic [13, 14]. However, considering the faster spread of the Delta variant, to contain the Guangzhou outbreak quickly and reduce economic and social costs and burdens, Guangzhou adopted “hierarchical and classified prevention and control (HCPC)” strategies based on the optimized and adjusted NPI measures, including mass testing, travel restrictions, extensive contact tracing, epidemic control with health code, and graded and adjusted management of communities according to risk levels, and the outbreak was contained quickly within 2 weeks. In our study, we mathematically simulated and projected transmissions based on this cluster by altering the timing and scaling of these measures, aimed at verifying the effectiveness of these policies on the Delta variant.

## Methods

### Data Source

According to the requirements of Prevention and Control of COVID-19 in China, positive cases will be directly reported online through the China Disease Control and Prevention Information System within 2 h. A field research report, which includes personal basic information, PCR-positive test result and clinical data, exposure history, and contact history, is needed for every positive case. We extracted the epidemic data from the field research report of COVID-19 cases in Guangzhou. The data collected and analyzed included the date of symptom onset (headache, fatigue, cough, hyposmia, fever, and other respiratory symptoms), history of close contact with confirmed cases, and real-time reverse transcriptase—polymerase chain reaction (RT-PCR) laboratory confirmation date. All identifiable personal information or controlled information were removed and not applicable in this study. The population size in each stratum was extracted from the Bulletin of the Seventh National Census of Guangzhou [15]. The ethical approval thus was waived by the Guangzhou Center for Disease Prevention and Control (CDC).

### The Hierarchical and Classified Prevention and Control Measures to the SARS-CoV-2 Delta Variant in Guangzhou

During this epidemic control, the Guangzhou CDC adopted “hierarchical and classified prevention and control measures” strategy and customized it as in Table 1.

**Table 1:** The hierarchical and classified prevention and control strategy in Guangzhou

Risk level	Classified management policy	Specification	
		Individual	Public
High	Closed-off management	Residents ordered to self-isolate and quarantine at home and not go out SARS-CoV-2 nucleic acid testing conducted 5 times on the 1st, 4th, 7th, 10th, and 14th day of the quarantine	Community lockdown, with one entrance open; residents allowed entrance into but no exit from isolated communities No vehicles allowed All business and trading service suspended, all entertainment, dining, shops and sports facilities suspended Students and childcare facilities closed, off-line teaching at kindergartens followed, and activities at training institutions shall be suspended
Medium	Closed and controlled management	Residents can only enter but cannot exit, and gatherings are strictly prohibited Nucleic acid tests shall be conducted total 2 times; after getting negative results, residents can go to supermarket, collect delivery and have other non-gathering activity	All communities keep only one entrance, and people can only enter but cannot exit All vehicles are not allowed All business and trading services as well as entertainment venues, restaurants, shops and sports facilities shall be suspended Keep one or two supermarkets/fresh markets on business, their staff shall not leave the controlled area and must wear mask properly when working Student and child care institutions shall be closed, off-line teaching at kindergartens followed, and activities training institutions shall be suspended
Low	Closed-loop management	People shall not go out unless it is necessary, keep close loop of "home-working place"	At the entrance/exit of the communities, follow the principle of “check body temperature, wear mask, scan the pass code, show the health code” Unnecessary personnel and vehicles are not allowed Public transportation shall conduct passenger limit at 60% of the maximum capacity Dining-in shall be banned at all restaurants Activities at entertainment venues and gathering places shall be suspended Supermarkets and fresh markets shall strengthen management of passenger flows and keep good ventilation Student and child care institutions shall be closed, off-line teaching at kindergartens followed, and activities training institutions shall be suspended

### Model Simulation

**Fig. 1:**
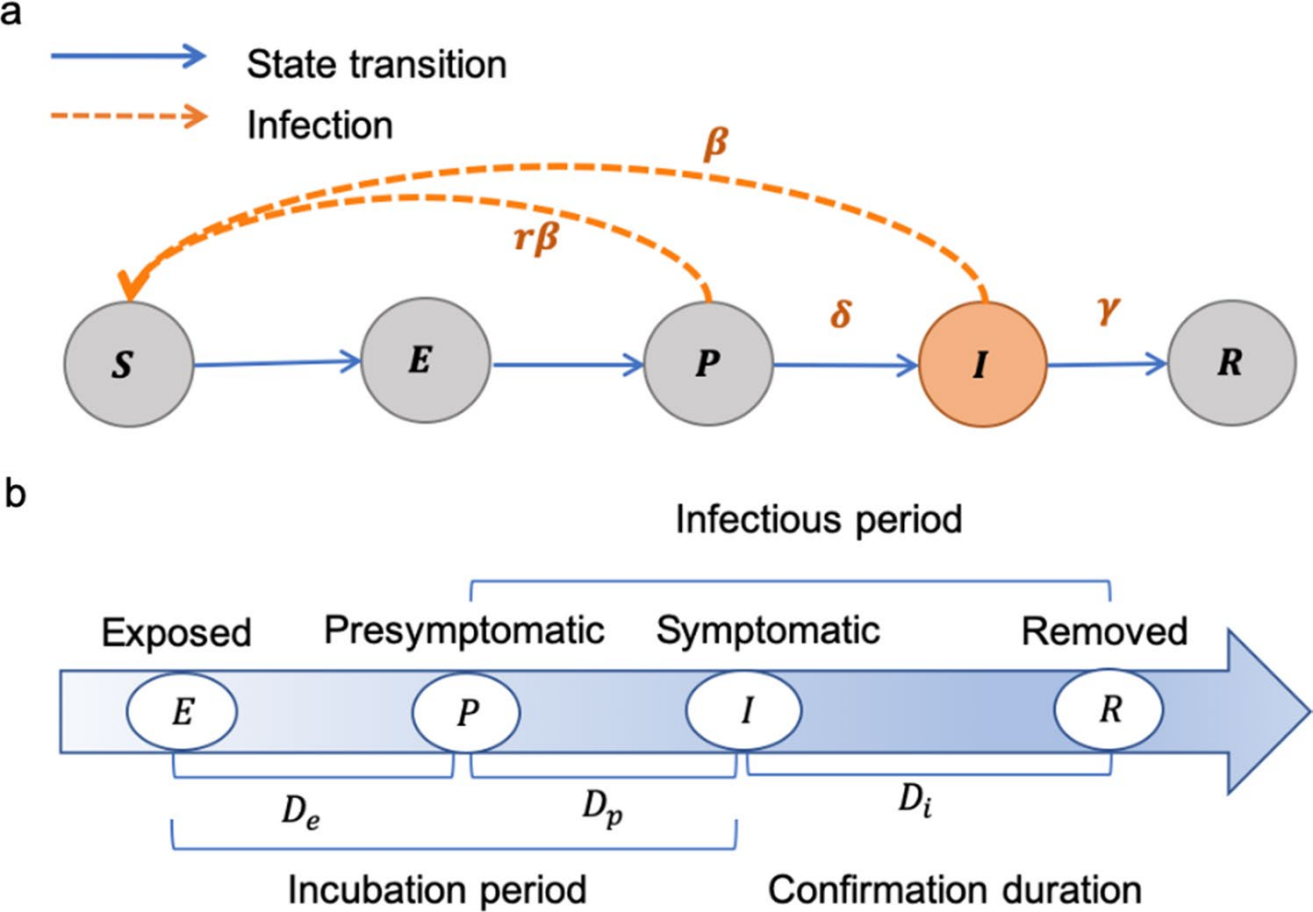
llustration of the SEPIR model. **a** We extended the classic four compartments of the SEIR model to five, including a new pre-symptomatic infectiousness (*P*) state. Relations among the compartments are shown with arrows. The direction of transition is indicated with solid blue arrows and the direction of infection with yellow dashed line arrows. The parameter $\upbeta$ would be variable across time periods. **b** Schematic disease course of cases. ${D}_{e}$, ${D}_{p}$ and ${D}_{i}$ are the time frame between different sates indicated. We assumed state *P* and *I* to be infectious in the overall course

Transmission dynamic models have been widely used in epidemic research [16–18] and their theoretical foundations and assumptions have been extensively tested and evaluated. By incorporation of the pre-symptomatic infectiousness (*P*) state, representing the infectiousness before symptom onset of SARS-COV-2 into the classic susceptible–exposed–infectious–recovered (SEIR) model, we developed a modified susceptible–exposed–pre-symptomatic–infectious–recovered (SEPIR) model. In summary, the SEPIR model includes the following compartments—susceptible (S): individuals who are susceptible to infection and have not yet been infected; exposed (E): individuals who are infected, but are not yet infectious; pre-symptomatic (P): individuals who are infected, but have not yet developed symptoms; infectious (I): individuals who are infected and are capable of transmitting the disease to others; recovered (R): individuals who have recovered from the disease. The rate of transmissibility $r$=0.55 between the state of* P* and *I* according to a previous study (Fig. 1) [19]. Further details can be found in reference [20].

To understand the effectiveness of policy deployment by Guangzhou during this outbreak control, we adjusted the timing of the policy deployment in our model simulation, as in reality different policies were implemented sequentially during May 18th–25th, May 26th–28th, May 29th–31st, and June 1st–18th (Table 2), where the designated ${\beta }_{1}$, ${\beta }_{2}$,${\beta }_{3}$, ${\beta }_{4}$ are shown in Table 3.

**Table 2:** Dates and segmented policy implementation

Date	Affected districts in Guangzhou	Policies
May 26th	Liwan	Crowd monitoring of high-risk area
May 29th	Liwan, Yuexiu, Haizhu, Baiyun, Fanyu	Communities of Hailong, Baihedong, Zhongnan, Dongjiao, and Chongkou streets locked down. Residents were allowed to move freely within communities
June 1st	Liwan, Haizhu, Fanyu	Communities of Zhongnan, Baihedong, Chongkou, Hailong, and Dongjiao streets were ordered to self-isolate at home

**Table 3:** Parameters for modeling

Parameters	Indication	May 18th–25th	May 26th–28th	May 29th–31st	June 1st–18th
$\beta$	Transmission rate	${\beta }_{1}$	${\beta }_{2}$	${\beta }_{3}$	${\beta }_{4}$
*De*	Latent period	2.1	2.1	2.1	2.1
${D}_{p}$	Pre-symptomatic period	2.3	2.3	2.3	2.3
${D}_{i}$	Infectious period	1.4	1.4	1.4	1.4
${T}_{g}$	Generation time	3.7	3.7	3.7	3.7
*N*	Population size	18,676,600	18,676,600	18,676,600	18,676,600

In addition, we calculated the incubation time 4.4 (95%CI.1.05–8.84) days and the infectious period 1.4 (95%CI. 0.20–5.01) days, by fitting the statistical data from epidemiological report to Weibull and Gamma distribution, respectively. The infectious pre-symptomatic period is 2.3 days as previously reported [21, 22] (Table 3).

The effective reproduction number is calculated as ${R}_{t}= \beta r{D}_{p}+{ \beta D}_{i}$ [20]. The initiation of simulation was set on May 18th, 2021 with the parameters indicated in Table 4.

## Results

### Basic Information

As of June 18, 2021, there were in total 153 reported cases in Guangzhou (146 confirmed cases, 7 asymptomatic cases). The first patient was infected on May 18th, and there was a peak of reported case during May 29 to June 5 with a total of 86. In general, there were 127 cases in Liwan District, 8 in Haizhu District, 10 in Nansha District, 4 in Panyu District, 3 in Baiyun District and 1 in Yuexiu District. In Liwan District, 89 cases were reported in Baihedong Street and 30 cases in Zhongnan Street, which are 57.62% and 19.87%, respectively, of the total number of Guangzhou cases. The age distribution of these patients were from 1 to 94 years, and the average age was 47.31 years. Patients under 18 years old comprised a proportion of 18.30% (28 cases), while patients more than 60 years old comprised 34.64% (53 cases). Most of them were retired people, housekeeping staff, unemployed people, and students, with a proportion of 32.03% (49 cases), 18.3% (28 cases) and 16.34% (25 cases), respectively.

**Table 4:** The initiation conditions for epidemic modeling

States	Indication	Initial value	Calculation
*S*	Susceptible individuals	18,676,596	*N-E0-P0-I0-R0*
*E*	Exposed individuals	2	Cumulative total cases during the time frame: *(Day0* + *Dp)–(day0* + *De* + *Dp)*
*P*	Presymptomatic individuals	0	Cumulative total cases during the time frame: *Day0–day0* + *Dp*
*I*	Infected individuals	1	Reported case on May 18th
*R*	Removed individuals	0	No removal on May 18th

### Validation of Our Model Simulation

**Fig. 2:**
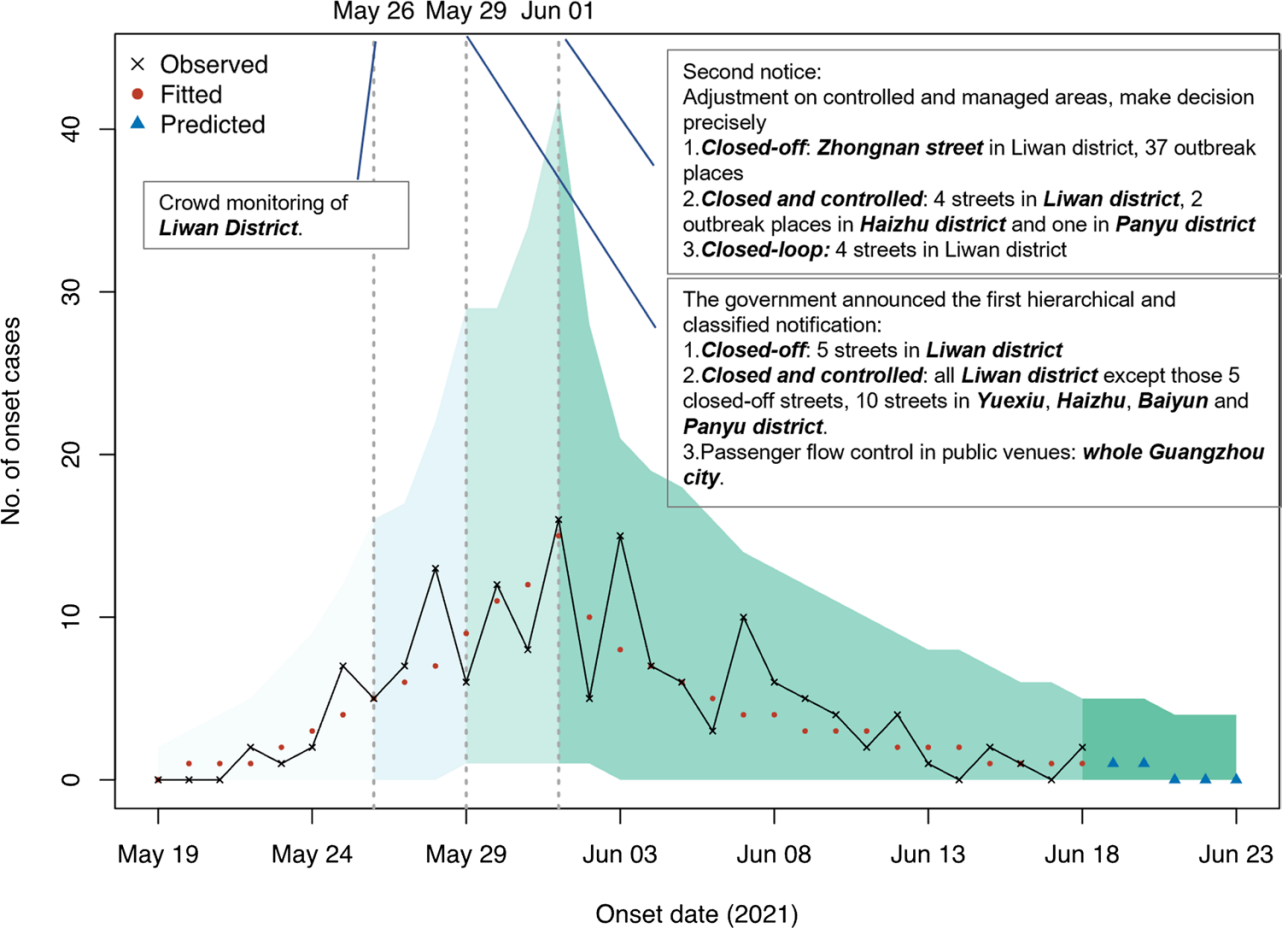
Daily cases were simulated between May 19th and June 23rd. The observed cases were plotted with a black cross. The fitted cases before June 18th and the predicted cases from June 19th are indicated with red dots and blue triangles. The vertical dotted lines label the three time points of policy implementation changes

To validate our modeling, we first simulated the development of the epidemic. The parameters associated with this outbreak were estimated with the Markov chain Monte Carlo (MCMC) in the R package BayesianTools (version 0.1.7) and analyzed with Gelman and Rubin's convergence diagnostic with the convergent psrf 1. Thus, there is a good agreement between our simulations for the daily onset cases and cumulated total cases as well as for the date of ending the epidemic (Fig. 2).

**Fig. 3:**
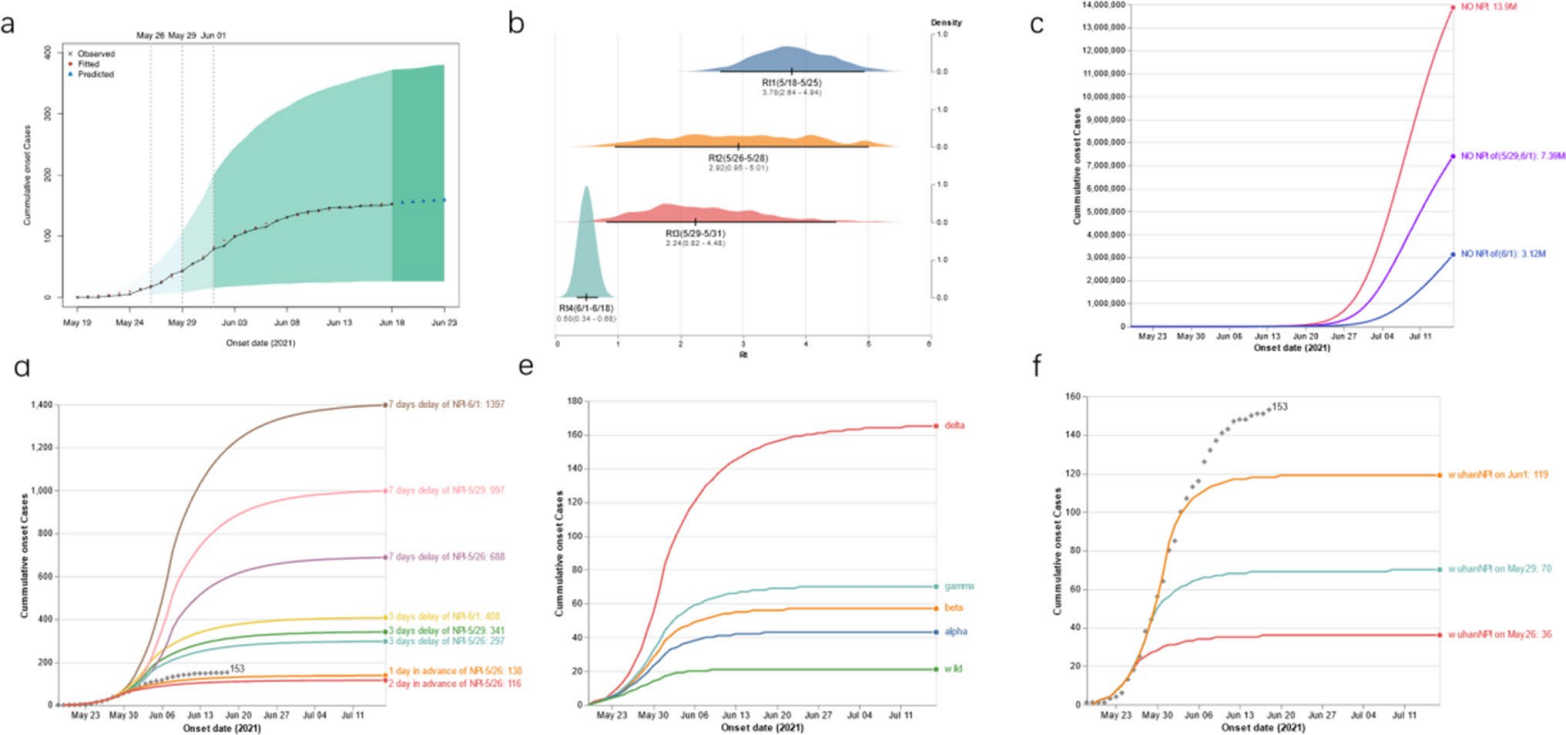
Simulation of the cumulative cases with our segmented strategy. **a** Reported and simulated cases from May 29th and June 23rd with three segmenting dates, May 26, May 29th and June 1st indicated on the plot. **b** Estimation of the effective reproduction number (Rt). The estimated Rt density distribution of each segmented time frames was plotted and aligned with each confidence interval. **c** Simulations of the transmission if NPIs were not deployed on specified dates. **d** Simulations of the transmission with adjusted policy timing. The dotted line is the actual case number reported. The solid lines are simulations with indicated timing on the right. **e** Simulation of the cumulative cases if infections were from different variants. The viral variants are labeled on the right of the plot. **f** Simulation of the cumulative cases if NPI implemented citywide lockdown

As the implementation of policies were largely segmented into four groups impacting the reproduction number (*R*_*t*_), we first estimated *R*_*t*_ as 3.78 (95%CI. 2.64–4.94), 2.92 (95%CI.0.95–5.01), 2.24 (95%CI.0.82–4.48) and 0.50 (95%CI.0.34–0.68), which showed a steady decrease compared with the previous segment of 22.75%, 23.29% and 77.68% (Fig. 3a). The *R*_*t*_ fell below 1 during segment 4, in agreement with the reality that the epidemic is under control and cleared quickly.

### Model Simulation on Policy Implementation

To understand the importance of the appropriate intervention, we first simulated scenarios in which different policies were not deployed. We found that if no active action was implemented on May 29 or June 1, there would be 7.39 or 3.12 million infections by July 17 (Fig. 3c). In addition, there would be 13.9 million infections if there was no preventive at all, indicating that active intervention is important for epidemic control.

### Model Simulation on the Timing of Implementation

It is generally agreed that early intervention, than late, would greatly affect the development of an epidemic. Here, we tried to understand the impact by adjusting the timing of simulated policy implementation by postponing for 3 days or 7 days the policies deployed on May 26th, 29th or June 1^st^; additionally, we also simulated 1-day or 2-day earlier implementation of the policies deployed on May 26th (Fig. 3d). Compared to reality, a 7-day delay of the implementation of policies on June 1st, May 29th and May 26th would result in an increase of the total cases by about 813.07%, 551.63% and 349.67%. The 3-day delay of the three policies on June 1st, May 29th and May 26th would cause the cumulative number of cases to increase by 166.67%, 122.87% and 94.12%. The 1-day or 2-day earlier implementation of interventions would result in 9.80% or 24.18% reduction of total infections.

The result showed that although there was little impact of the earlier implementation of the policies deployed on May 26th, the impact of delayed policy implementation may result in catastrophic outcomes of the epidemic, supporting the notion that an appropriate and a good timing of policy implementation is essential for transmission control.

### Model Simulation of the Introduction of Different Viral Variants

SARS-CoV-2 is a continuously evolving RNA virus. The transmissibility differs greatly among different viral variants. Here, we further assumed and analyzed the scale of transmission if a different viral variant was introduced instead of the delta variant (Fig. 3e). Adjusted with the vaccination rate of ~ 40% for the population and the reported *R*_*0*_ of different viral variants (Table 5) [23, 24], we calculated the $R^{\prime}t$ of each scenario with equation $R^{\prime}_{t} = R_{t} *\left( {1 - VE*0.4} \right).$ The data show that the cumulative case numbers by June 18th would be 70, 57, 43 and 21, that is, 54.25%, 62.75%, 71.90% or 86.27% reduction for Alpha, Beta and Gamma variants than the Delta variant, indicating that the current strategies are still effective to contain the known variants.

**Table 5:** Parameters for SARS-CoV-2 variants simulation

Variants	*R*_*0*_	Vaccine effectiveness	Ratio of infectivity compared to Delta variant
Alpha (B.1.1.7)	3.3	70%	0.66
Beta (B.1.351)	3.2	46%	0.73
Gamma (P.1)	3.5	49%	0.78
Delta (B.1.617)	5	65%	1
Original variant	2.5	70%	0.5

### Model Simulation on More Stringent Citywide Lockdown

It is noteworthy that, compared to a more stringent citywide lockdown, the Guangzhou CDC implemented very precise and timely updated interventions during this epidemic. To evaluate the impact of this strategy, we simulated the scenario with a citywide lockdown referring to the evidence reported the Rt reduced 91.57% before and after the City of Wuhan lockdown [20]. Thus, we simulated the citywide lockdown for May 26th, May 29th and June 1st with 91.57% reduction of Rt, and simulated the cumulated total case numbers as 36, 70 and 119, that is 76.47%, 54.25% or 22.22% reduction of the actual cases in reality (Fig. 3f).

## Discussion

With the stringent border control and steady rolling out of vaccination from the beginning of 2021, China has become one of the countries that maintains no large-scale community transmissions after the successful containment of the first wave of COVID-19, although several sporadic clusters initiated likely through imported routes were reported [25–30]. However, from May to July 2021, there have been several multi-point outbreaks caused by the Delta variant of SARS-COV-2 in China, alerting that the transmission and prevalence of the Delta variant of SARS-COV-2 will continue in the next time period. We should actively consider and adjust the intensity of the prevention and control measures. In this study, we used an SEPIR model to simulate the Delta variant transmission and the effectiveness of the HCPC strategy for a recent cluster in Guangzhou, China. The outbreak studied here is the first local transmission of the Delta variant in China. Our results suggested that the HCPC strategy was able to effectively reduce the scale of transmission, shorten the epidemic period and reduce the total number of infections. The lesson learned and the experience gained during the transmission containment are useful for battling against SARS-CoV-2 around the globe.

Based on our simulation, the outbreak would become out of control, with 7 million estimated infections under the assumption of the absence of any interventions than the 153 reported cases in reality in Guangzhou. The simulation on delayed implementation of interventions showed that the total case number would also surge greatly if the interventions were delayed by days (3–7 days).

It is noteworthy that the Guangzhou CDC adjusted the containment strategies based on the real-time analysis of the up-to-date epidemic. The hierarchical and classified prevention and control measures were effective to cut the community transmission of the Delta variant in the city. A reduction of 99% infections was predicted if a delay happened compared to the number in reality, supporting the notion that early implementation is essential for COVID-19 containment, also in agreement with the conclusion in a study by Islam et al. based on a meta-analysis for 149 countries globally [31]. In addition, a model simulation by Oraby and Brauner et al. on the effectiveness of government-driven interventions concluded that timely adjustment of NPIs is essential as an effective strategy for COVID-19 containment [32, 33].

In this study, we also simulated and analyzed the impact of the most stringent citywide lockdown implemented during the beginning of the pandemic in Wuhan. Our data suggested that although the implementation of citywide lockdown would reduce the scale of the outbreak to 119 cases, a 22.22% reduction than the 153 real-world cases reported in Guangzhou during the outbreak, we would predict that the hierarchical and classified prevention and control measures are economically cost-effective. Wuhan reported 4.7% of GDP losses, about 2.3% GDP losses per month during the 2-month citywide lockdown (Wuhan Bureau of Statistics data [34]). Thus, we simply predicted that implementation of 1-month lockdown would also result in a 2.3% reduction of the total GDP in Guangzhou, estimated as 48.3 ~ 52.9 billion Chinese yuan. However, implementation of the hierarchical and classified prevention and control measures may lead to a better outcome compared to the strategy of citywide lockdown, that is, a 0.5 ~ 2.0% GDP losses, or estimated as 10.5 ~ 23 billion Chinese yuan. In addition, although there was a reduction of 34 infections between the simulation and reality, the duration of the outbreak was modeled to end on June 15th as well, similar to reality, indicating that the hierarchical and classified prevention and control measures can be effective to contain the transmission within a maximum incubation period of 14 days to end the outbreak. Although previous modeling had shown that the citywide lockdown taken during the Wuhan outbreak in 2020 reduced infections by 96% [20]. J. Dehning et al*.* and N. Banholzer et al*.* found that in cities that implemented measures such as closing educational institutions and banning gatherings, the effect on epidemic prevention and control was very small [35, 36]. Additionally, Brauner J M et al*.* found that it was possible to reduce Rt to below 1 without citywide lockdown [33]. As social cost is an essential element in consideration during an epidemic control, the hierarchical and classified prevention and control measures are more practical to balance both purposes during their application.

To further evaluate the effectiveness of the intervention against different variants of concerns, we simulated transmission scenarios with four known variants. The results showed that the transmission scale of Gamma, Beta, Alpha and the wild-type variants all showed more than 50% reduction compared with the actual cumulative infections with the Delta variant, supporting that the current strategies are effective to contain previously or currently circulating major variants.

We found that timely and more precise multiple interventions including mass testing and contact tracing of at-risk populations are effective in controlling COVID-19 outbreak. Similar outcomes were found in New Zealand [37], Singapore [38], Hong Kong [39], and other cities of Mainland China [40], when these countries or region adopted transitional “COVID-zero” strategy to get more time to improve the COVID-19 vaccination rates. Although there has been no consensus about the best strategy for managing COVID-19 outbreaks due to differences in politics, culture and economy between countries, we provide an effective controlling strategy that requires greater government enforcement and citizen compliance.

### Limitations

The SEPIR model provides an insight into the spread of the disease and helps to evaluate the effectiveness of control measures in reducing the spread of the disease. The results from transmission dynamic models can inform policy decisions related to the COVID-19 pandemic, such as when to implement or lift restrictions. This model also has some limitations. First is the assumptions and uncertainties: the transmission dynamic models rely on many assumptions about disease transmission, such as the infectivity of the virus and the effectiveness of control measures. These assumptions may introduce uncertainties in the model's predictions. Second is the difficulty in modeling complex situations: Modeling complex scenarios, such as the impact of multiple control measures or the emergence of new virus variants, can be challenging and may require more complex models. Third, in our simulations, we assumed that resources were adequate and all the NPIs were effectively implemented even as the numbers of cases surged. In reality, as disease transmission increased, some resources (e.g., centralized quarantine facilities) would be depleted and eventually become short. Fourthly, though HCPC is an integrated strategy with multiple interventions, our study could only quantify a combined effect rather than the contribution of each intervention.

## Conclusions

In summary, the HCPC strategy can effectively contain COVID-19 transmission caused by the Delta variant. Under such strategies, large-scale mass testing and effectively graded and adjusted management of affected areas were the top selections as major policies and are thought to contribute to the successful containments of several outbreaks from expanding to larger scales [41–43]. In addition, through mathematic modeling, we suggested that it is important to apply intervention in a timely manner. This is helpful not only as a means to supplement the current COVID-19 epidemic prevention and control strategy in China, but also provides potentially valuable supplement to COVID-19 outbreak prevention and control worldwide. As the world is faced with the serious challenges of emerging and reemerging infectious diseases, the HPCP strategy provides valuable experience for other countries to deal with the local epidemic breakouts caused by an emerging infectious pathogen such as SARS-CoV-2 Delta. For the future, our study also proposed a feasible approach to conduct a reliable assessment of prevention policy before implementing intervention for emerging infectious diseases.

## Data Availability

The data that support the findings of this study originate from Guangzhou Center for Disease Control and Prevention. Case data are derived from epidemiological investigation reports that are not publicly available.

## Abbreviations

HCPC: Hierarchical and classified prevention and control measures
CDC: Center for Disease Prevention and Control (CDC)
CI: Confidence interval
COVID-19: Coronavirus disease 2019
MCMC: Markov chain Monte Carlo
NPI: Non-pharmaceutical intervention
SEIR: Susceptible–exposed–infectious–recovered
SEPIR: Susceptible-exposed–pre-symptomatic–infectious–recovered
